# Bioclogging in porous media: influence in reduction of hydraulic conductivity and organic contaminants during synthetic leachate permeation

**DOI:** 10.1186/s40201-014-0126-2

**Published:** 2014-10-29

**Authors:** Subramaniam Kanmani, Rajan Gandhimathi, Kasinathan Muthukkumaran

**Affiliations:** Department of Civil Engineering, National Institute of Technology, Tiruchirappalli, Tamilnadu, India

**Keywords:** Biofilm, Hydraulic conductivity, Microbial culture, Synthetic leachate

## Abstract

In this study the concept of biofilm accumulation in the sand column was promoted to assess the changes in hydraulic conductivity and concentration of organic contaminants of the synthetic leachate. Four different combinations of column study were carried out using synthetic leachate as a substrate solution. Mixed and stratified mode of experiments with two different sizes (0.3 mm and 0.6 mm) of sand grains were used for column filling. Two columns were acting as a blank, the remaining two columns amended with mixed microbial cultures which were isolated from leachate. The column was operated with continuous synthetic leachate supply for 45 days. The results indicated that the highest hydraulic conductivity reduction occurred in the mixed sand microbial column with 98.8% when compared to stratified sand microbial column. The analysis of organic contaminants of the effluent leachate was also clearly shown that the mixed sand amended with microbes poses a suitable remedial measure when compared to natural and synthetic liners for controlling the leachate migration in the subsurface environment.

## Introduction

The generation of solid waste has become an increasing environmental and public health problem everywhere in the universe, especially in the developing countries [1]. Open dumps are the oldest and the most common mode of disposing of solid waste. Although in recent years, thousands have been closed, many still are being used [2]. The dumping of solid waste in uncontrolled landfills can have significant impacts on the environment and human health [3]. Leachate migrations from waste sites or landfills and the release of pollutants from sediment (under certain conditions) pose a high risk to groundwater resource if not adequately managed. Plenty of materials have been identified as contaminants of ground water. These include synthetic organic chemicals, hydrocarbons, inorganic cations, inorganic anions, pathogens, and radio nuclides. Most of these materials dissolve in water to varying degrees [4]. Their impact on groundwater continues to raise concern and have become the subject of recent and past investigations [5-15].

Continuously increasing awareness in preserving the groundwater supplies from contaminants generated from waste sites has given rise to the design of well-isolated containment structures. These measures generally involve the application of low permeability natural clays and sand-bentonite mixtures or synthetic materials [16]. Compacted natural clays are often used in constructing hydraulic barriers underneath waste containment systems. The hydraulic conductivity must be less than or equal to 1 × 10^−7^ cm/s for soil liners and covers used to contain hazardous waste, industrial waste, and municipal waste [17]. In the absence of impervious natural clay liners, Geosynthetic Clay Liners (GCLs) are progressively being employed as constituent of a composite liner with geomembranes (GM) in landfill barrier systems [18]. The primary advantages of the GCL are the limited thickness, the good compliance with differential settlements of the underlying ground or waste, easy installation and low price. On the other hand, the limited thickness of this barrier can produce: (1) vulnerability to mechanical accidents, (2) limited sorption capacity, and (3) an expected significant increase of diffusive transport if an underlying attenuation mineral layer is not provided [19]. Exothermic degradation of organic matter or hydration of incinerator ash within the landfill generates heat inside the waste pile. This creates thermal gradients through the composite liner, which hold the potential to cause a net movement of moisture away from the warmer liner. The result is a potential for desiccation that may impair the long-term performance of the GCL [20]. As the role of the GCLs broadens, they are being investigated intensively, particularly in respect to their hydraulic and diffusion characteristics, chemical compatibility, mechanical behavior, durability and gas migration [21-30]. These methods have proved to be expensive and, in many cases, ineffective at achieving the proposed level of cleanup. The biofilm accumulation in the porous media is an effective way in the reduction of hydraulic conductivity and concentration of organic contaminants from the leachate. This technique stimulates the microbes to remove subsurface pollutants, which is prominent because it has the potential to: permanently eliminate the contaminants through biochemical transformation or mineralization; avoid harsh chemical and physical treatments; operate in situ and be cost effective [31,32]. Aquifer material is excavated and replaced with microbial cultures as horizontal treatment layers and as vertical treatment walls. These engineered biofilm layers eliminate the contaminants on in-situ transformations [33]. Among the various contaminated groundwater remediation measures that consider results, risks, and costs, biofilm accumulation is preferred [34].

A biofilm is a well organized, cooperating community of microorganisms. Microbial cells attach to the surfaces and develop a biofilm. Biofilm associated cell is differentiated from suspended counterparts by reduced growth rate, upwards and down regulation of gene and generation of extra polymeric substances [35]. Genetic studies have revealed that biofilms are formed through multiple steps. They require intracellular signalling and transcribe different set of genes from planktonic cell [36]. Biofilm accumulation in porous media is the overall result of microbial cell adsorption, desorption, growth on surfaces, detachment and filtration. The microbial growth in land and the resultant decrease in hydraulic conductivity are much connected with groundwater recharge, wastewater, soil injection, enhanced oil recovery schemes, and the in situ bioremediation of organic contaminants in the subsurface environment [37].

A turn of previous experiments have looked into the issue of microbial biomass growth on reduction of porosity, permeability and hydraulic conductivity in porous media [38-41,37,42,43] and rock-fracture analogues [41,44] which could induce an outcome on the fate of contaminants in the subsurface.

Seki *et al.* [45] concluded that bacterial clogging proceeds more rapidly than fungal clogging probably because bacteria grow faster than fungi on the ground surface. Taylor and Jaffe [38] conducted experiments on sand packed column reactors to study the issue of biomass growth on soil permeability and dispersivity using methanol as a growth substrate. From the answers, the permeability reduction was noted by three orders of magnitude. Vandevivere and Baveye [40] and Bielefeldt *et al.* [46] estimated the hydraulic conductivity reductions of three orders of magnitude. Cusack *et al.* [47] reported that the decrease in K (hydraulic conductivity) was achieved as 99% in sandstone by using starved bacteria. Cunningham *et al*. [39] used sand (grain size: 0.54 mm and 0.12 mm) column inoculated with bacteria and applied a constant head difference between inflow and outflow. A reduction of more than 90% in hydraulic conductivity and 50–90% in porosity was observed. Brough *et al*. [48] noted a reduction of hydraulic conductivity of between 28 and 79% using 35 series of column experiment.

Kim [49] evaluated the changes in hydraulic conductivity as 1 × 10-4 cm/Sec is using sand columns (sand grains of 0.25 millimeter to 0.42 mm in size) due to barrier formation. Kim et al. [37] reported the hydraulic conductivity reduction by 1/8000 of the initial hydraulic conductivity when the uninterrupted provision of substrate and oxygen. Zhong and Wu *et al.* [34] investigated the bioclogging in porous media (sand grains of size 0.2 mm to 0.5 mm) under continuous flow condition and achieved the hydraulic conductivity reduction in one order magnitude. Several authors reported a significant reduction of hydraulic conductivity due to bioclogging. These studies demonstrated that the biobarrier may be a promising technology for containing contaminant plume in the field. Improved understanding of these interactions will lead to industrial and environmental applications in bio hydrometallurgy, enhanced oil recovery, and bioremediation of contaminated groundwater and soil [39].

In this research study, the influence of biofilm accumulation using sand column is described by (1) the reduction in hydraulic conductivity of the inoculated microbial sand column (2) the physico-chemical characteristic of the effluent leachate such as pH, turbidity, total dissolved solids (TDS), oxidation reduction potential (ORP), nitrates, phosphates and degradation of organic contaminants in terms of COD.

## Materials and methods

### Sand

Sand used in this study was collected from a river shore Cauvery, which is located in the Trichirappalli district, Tamilnadu. The physical properties of the sand were determined as per the code IS 2720: 1987 [50] and are presented in Table 1. Sand thus collected were sieved, the portions which retained on 0.6 mm and 0.3 mm were washed with distilled water, cooled to room temperature and preserved in clean plastic containers for subsequent use. Sand samples were sterilized in an autoclave before microorganisms were inoculated.

**Table 1:** **Physical properties of sand**

**S. No**	**Properties**	**Value**
1.	Specific gravity	2.65
2.	Porosity	0.33
3.	Permeability (cm/sec)	1 × 10^−1^
4.	Dry density (g/cm^3^)	1.950
5.	Organic content (%)	0.177
6.	Moisture content (%)	0.049

### Chemicals

All the chemicals used in this study were of analytical reagent (AR) grade and were supplied by Merck specialities Ltd., Mumbai, India. Glassware used for analysis was washed with an acid solution followed by distilled water.

### Isolation of microorganism

In this study, bacterial strains were isolated from the leachate samples collected from an open dumping site at Ariyamangalam, Tiruchirappalli, Tamilnadu. To eliminate the target contaminants from the leachate, the experimental microorganisms were isolated from leachate sample according to normal microbiological procedures. The nutrient medium (NM) for bacterial growth consists of Peptone (10 g), Beef extract (2 g), Yeast extract (1 g), and Sodium Chloride (5 g) in 1 L of distilled water [51]. The pH was maintained at 7 ± 0.2 through the addition of HCl (0.1 N) or NaOH (0.1 N). The media were sterilized by wet autoclaving at 15 kPa and 121°C for 20 min. Approximately 10 ml of leachate was added to 100 ml of nutrient medium (NM) and incubated for 48 h at 37°C in facultative condition [52]. The shake flask cultures were closed using Teflon stoppers.

The growth rate of the microorganisms was estimated by measuring Optical Density (OD), defined as the logarithmic ratio of the initial light intensity to the light intensity not disturbed by the microorganisms. OD can be measured by using a spectrophotometer at 600 NM wavelengths [53]. A loopful of incubated mixed culture was streaked on agar slants, incubated for 24 h and stored in the freezer at 4°C for further use.

### Bacterial cultivation in synthetic leachate

The substrate solution used in this column study was synthetic leachate (Table 2) [54]. It consists of three volatile fatty acids with various salts and a trace metal solution. The physical and chemical characteristics of the synthetic leachate were analyzed as per standard methods [55] and are presented in Table 3. Synthetic leachate was made in bulk and refrigerated for further usage. Chemical Oxygen Demand (COD) of the synthetic leachate was measured at 14000 mg/L using the closed reflux method. 40 ml of isolated mixed bacterial culture was transplanted to 400 ml of synthetic leachate solution contains 14,000 mg/L of COD [52]. This culture was incubated aerobically at 27°C on a 125-RPM orbital shaker for 7 days. Aerobic conditions were utilized to quickly generate biomass for sand column experiments because the microbial species of interest are facultative [56]. During the microbial growth, the increase in MLSS concentration was assessed with the reduction in COD concentration. Steady state COD concentration was noted in seven days with the microbial count of 340 × 105 cells/cc. The developed microbial biomass solution was used for filling the experimental column.

**Table 2:** **Composition of synthetic leachate**

**S. No.**	**Component**	**Quantity per litre**
1.	Acetic acid	7 ml
2.	Propionic acid	5 ml
3.	Butyric acid	1 ml
4.	K_2_HPO_4_	30 mg
5.	KHCO_3_	312 mg
6.	K_2_CO_3_	324 mg
7.	NaCl	1440 mg
8.	NaNO_3_	50 mg
9.	NaHCO_3_	3012 mg
10.	CaCl_2_	2882 mg
11.	MgCl_2_6 H_2_O	3114 mg
12.	MgSO_4_	156 mg
13.	NH_4_HCO_3_	2439 mg
14.	CO(NH_2_)_2_	695 mg
15.	Na_2_S. 9H_2_O	Titrate to an Eh 120 mV:180 mV
16.	NaOH	Titrate to a pH 5.8–6.0
17.	Trace metal solution (TMS)	1 ml
18.	Distilled Water	To make 1 l
**Composition of trace metal solution (TMS)**		
1.	FeSO_4_	2000 mg
2.	H_3_BO_4_	50 mg
3.	ZnSO_4_.7H_2_O	50 mg
4.	CuSO_4_.5H_2_O	40 mg
5.	MnSO_4_.7H_2_O	500 mg
6.	(NH_4_)_6_Mo_7_O_24_.4H_2_O	50 mg
7.	Al_2_(SO)_3_.16H_2_O	30 mg
8.	CoSO_4_.7H_2_O	150 mg
9.	NiSO_4_.6H_2_O	500 mg
10.	96% concentration H_2_SO_4_	1 ml
11.	Distilled Water	To make 1 l

**Table 3:** **Physicochemical characteristics of synthetic leachate**

**S. No.**	**Parameters**	**Value**
1.	pH	6
2.	Turbidity in NTU	1
3.	Total Dissolved Solids (TDS) in mg/L	13000
4.	Nitrates (NO_3_^−^) in mg/L	3.5
5.	Phosphates (PO_4_^3−^) in mg/L	27
6.	Chemical Oxygen Demand (COD) in mg/L	14000

### Experimental setup

**Figure 1:**
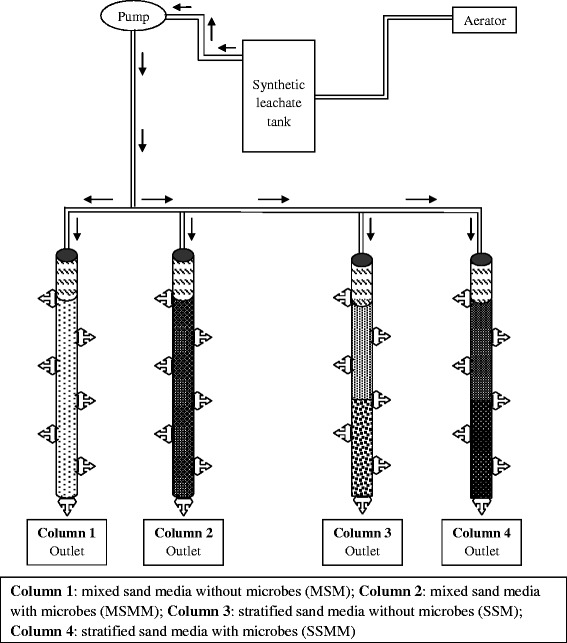
**Schematic representation of experimental setup.**

A column study was carried out to understand the changes in the reduction of hydraulic conductivity and organic contaminants of the effluent leachate due to the biofilm accumulation. Four identical columns made of glass with a diameter of 2.5 cm and a length of 35 cm was used for the study. Figure 1 shows the schematic representation of the experimental set-up. The column was fabricated using gas-leak-proof materials and parts. Synthetic leachate was supplied as a substrate solution throughout the study.

The cleaned sand of size 0.6 mm and 0.3 mm were used for filling each column. A mixture of 70% of 0.3 mm sand and 30% of 0.6 mm sand, by weight (70:30 mix) were used for filling the first two columns. Column 1 was filled with mixed sand media (MSM); whereas column 2 was amended with microbes in mixed sand media (MSMM). The other two columns were filled with stratified layer of sand media such as, 110 g of 0.3 mm sand in the top layer (0–15 cm) and 100 g of 0.6 mm sand in the bottom layer (15 – 30 cm). Column 3 was filled with sand media in stratified mode (SSM); whereas column 4 was amended with microbes in stratified sand media (SSMM). Totally, 210 grams of sand media were weighed and packed for a depth of 30 cm of each column. Column 1 and 3 were used as a blank, without any addition or inoculation. To inoculate the columns 2 and 4, 80 ml of developing a culture containing approximately 340 × 10^5^ cells/ml were mixed with 210 g of sterilized sand, and the resulting slurry was aseptically poured into the columns. The inoculated sand columns were incubated under no-flow conditions for 24 h, to promote bacterial attachment to the sand [57]. Glass wools were placed at the outlet of the each column to retain the sand in the columns. The experiments were carried out at a temperature of 20 ± 1°C and the sand-bed columns were side protected from the light [58].

The synthetic leachate described previously was fed to the inlet tank, which can be located at the preferred height. The overflow was circulated to the inlet tank to maintain a constant hydraulic head. The pH of the synthetic leachate was adjusted as 5.8 to 6.0 throughout the experimental study. A four channel peristaltic pump was set to distribute a constant flow of 1 ml/min synthetic leachate for a combined flow rate of 4 ml/min. volumetric flow rates were monitored frequently. To begin with, the outlet flow rate was also set at 1 ml/min for all the columns. Saturated conditions in the columns were kept by controlling the water surface 5 cm above the sand-bed.

The hydraulic conductivity was measured as a function of time in each column using the constant head method based on ASTM D 2434 [59]. Hydraulic conductivity was calculated using Darcy’s law, as follows:

1 $$ K=QL/A\times t\times h $$

Where,

K - hydraulic conductivity (cm/s)
Q - volume of flow (cm^3^)
L - length specimen (cm)
A - cross sectional specimen area (cm^2^)
t - time during which Q occurs (s) and
h - hydraulic head (cm)

The effluent samples were collected daily from the outlet port of the each column and the physicochemical parameters such as pH, turbidity, Total Dissolved Solids (TDS), Nitrates (NO_3_^−^), Phosphates (PO_4_^3−^), Oxidation Reduction Potential (ORP) and COD were analyzed as per standard methods [55]. All the analyses in this study were repeated two or three times until concordant values were obtained.

After completion of the experimental study, the sand samples were collected from each column and surface morphology has been visualized by Scanning electron microscopy (SEM). Since SEM is important for high resolution visualization of bacterial biofilms [60]. In SEM, biofilm specimens are prepared by fixation, staining, drying and conductively coating prior to imaging under high vacuum [61]. Air dried samples were spread out on the sample mounted on aluminum stab sequenced by coating with a thin layer of gold under vacuum to increase the electron conduction and to increase the quality of the images [62]. The scanning electron imaging of sand samples of each column after leaching was done at 1–15 KV uses a microscope equipped with a filled-emission cathode. The images were captured using SEM HITACHI (Model: S3000N) instrument with 100× and 500× magnification.

## Results and discussion

### Hydraulic conductivity (K)

**Figure 2:**
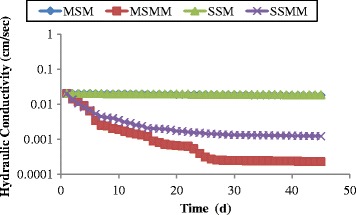
**Changes in hydraulic conductivity as function of time.**

Four different sets of experiments were conducted for assessing changes in hydraulic conductivity permeated with a synthetic leachate solution. A constant head was held in each column throughout the experiment and flow rates were measured volumetrically at 24 h intervals. From these data, together with column geometry, hydraulic conductivity values were computed using Darcy’s equation. The change in hydraulic conductivity as a function of time is shown in Figure 2. From the Figure 2, the discussions were made as follows: Initial hydraulic conductivity of the column was 2.03 × 10^−2^ cm/s. After 45 days the hydraulic conductivity was decreased as 1.82 × 10^−2^ (MSM); 2.31 × 10^−4^ (MSMM); 1.88 × 10^−2^ (SSM); 1.23 × 10^−3^ (SSMM) in the columns. The blank column (MSM and SSM) tests resulted in a very less decrease in hydraulic conductivity when compared to the microbial inoculated columns. The hydraulic conductivity in these columns was observed at almost similar as that of initial value for the first 15 days, later it started slight decrease due to precipitation of the permeated synthetic leachate [63].

After 45 days, the reduction in hydraulic conductivity was observed as 10% in MSM and 7% in SSM. The microbial inoculated column MSMM reduced hydraulic conductivity up to 98.88% during the 45 days of experimental operation. Within 2–3 days after microbial inoculation, a uniform biofilm of detectable thickness could be observed on the exposed edges of reactor media particles. Rapid decrease in K (68%) was attained in the first six days due to hasty microbial growth and its uniform biofilm thickness along the column. Mixed bio culture in the porous media and the uniform biofilm formation are responsible in hydraulic conductivity reduction from 24^th^ day onwards. The uniformity biofilm growth was happening due to the mixed proportion (70:30 mix) of porous sandy media; it allows the microbes to attach with the sand particles and further increases the biofilm growth. Hydraulic conductivity in the stratified media inoculated with microbes achieved a 93.94% reduction after 45 days of operation. For the first 6 days, 66% of reduction in K was observed due to the initial inoculums in the column. Subsequently, 90% of K reduction was achieved on 19th day, due to the biofilm formation. During the period between 27 and 45, the K value stabilized at 93%. The higher reduction in hydraulic conductivity was achieved in MSMM column when compared to SSMM column.

### Effluent leachate composition changes

**Figure 3:**
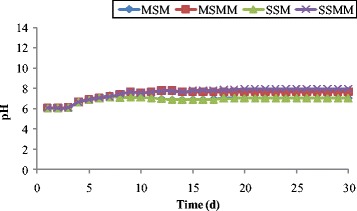
**Changes in pH as function of time.**

Effluent leachate samples were collected from the outlet of the each column and tested for pH, turbidity, TDS, ORP, Nitrates, Phosphate and COD concentrations on a daily basis. The influent pH was maintained between 6 and 6.05 during the experimental study. The change in pH as a function of time is shown in Figure 3. From the Figure 3, it is observed that the pH of the effluent slightly increased than the influent pH (ranged 6 – 7.01) in the blank columns (MSM, SSM) during the study. The effluent of blank column is in acidic nature, due to the presence of volatile acids in the supplied influent leachate. Similar effluent pH values were reported from column studies performed using synthetic leachate [64,65]. While in the microbial inoculated column, the pH range varied between 6 and 8. In the column MSMM, there was a gradual increase in effluent pH from 6 to 7.65. Equally, in the column SSMM also, the value of pH gradually turns to alkaline. This may be attributed to the decrease in the concentration of free volatile acids due to anaerobic decomposition, as fatty acids can be partially ionized and contribute to higher pH values [66].

**Figure 4:**
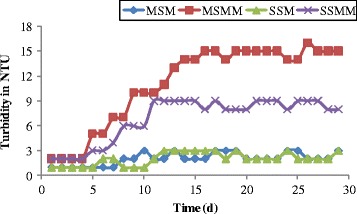
**Changes in turbidity as function of time.**

Turbidity is an indirect measure of biological activity, it consists of microorganisms and suspended and colloidal material generated by biological activity [67]. The initial turbidity (1 NTU) of the supplied influent synthetic leachate was stable throughout the experiment. The changes in the turbidity as a function of time for each column are shown in Figure 4. From the Figure 4, the initial turbidity is found to be constant for the first 5 days in blank columns (MSM, SSM) and, it increases slightly up to the maximum of 3 NTU in the effluent during the study period. The turbidity levels were observed as significantly higher in effluent leachates in the microbial inoculated columns. The increase in the turbidity of the effluent leachate over time was indicative of biomass growth and accumulation in the sand-bed columns [58,67]. Higher turbidity value was observed as 16 NTU in MSMM and 9 NTU in SSMM due to the presence of microbial activity.

**Figure 5:**
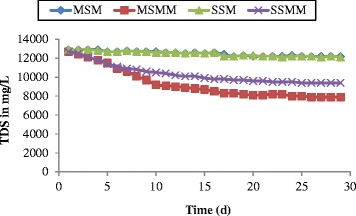
**Changes in TDS as function of time.**

The total dissolved solids were measured in the effluent leachate on a daily basis from each column and it is shown in Figure 5. The TDS concentration of influent leachate was constantly maintained as 12900 mg/L, throughout the experimental study. Very less reduction in TDS concentration was noted in the blank columns due to very less development of microorganisms. In the microbial columns, the mixed sand media allows producing extra polymeric substances in the gap formed between irregular sizes of grains. Thus, the developed microbes were completely utilised the organic nutrients supplied by the influent leachate and reduced more TDS concentration in the effluent leachate. The less reduction in TDS concentration of stratified column indicates that, less biofilm growth due to the uniformity sizes of coarse sand grains filled inside the column. There is a chance of a detachment of microbes in the coarse layer due to the more space between the sand grain particles.

**Figure 6:**
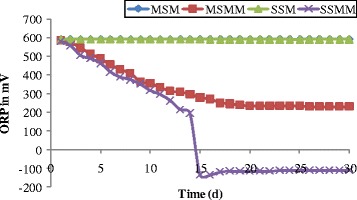
**Changes in ORP as function of time.**

ORP can be a useful parameter to check the digester performance since it measures the net value of all complex oxidation-reduction reactions within an aqueous environment. The measurement of ORP has been used in several instances as an environmental parameter in anaerobic digestion systems [68-71]. The changes in ORP were studied in the effluent leachate from each column, which is shown in Figure 6. From the Figure 6, the measured ORP values in the blank columns changes in the range between 594 mV and 590 mV. In the column MSMM, the ORP value was observed clearly as 230 mV at 18^th^ day and it was maintained throughout the experimental operation. The stable value of ORP indicates the constant growth of microorganisms and even distribution of biofilm layer inside the column [67,72]. In the stratified sand column (SSMM), the ORP value was decreased gradually from the initial value. At 15^th^ day of operation, the ORP changes from +150 mV to −150 mV due to anaerobic creation inside the column at pH 7.9. The report of Metcalf and Eddy [73] clears that, the dramatic decrease in the ORP value (+150 mV to −150 mV) at a single day, may be due to the dissolved oxygen concentration decline in the column. The ORP value was maintained as stable as −150 mV throughout the remaining days of experimental condition. This may be due to the initial stage of the propionic acid fermentation process in the column. An ORP of more than −150 mV always led to propionic acid-type fermentation at any pH and, at a pH of about 5.0, either propionic acid or butyric acid fermentation might occur depending on whether the ORP value is high or low [74,75].

**Figure 7:**
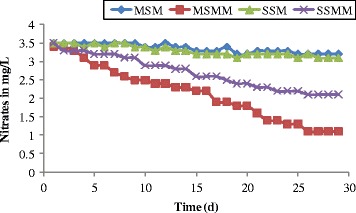
**Changes in nitrate as function of time.**

**Figure 8:**
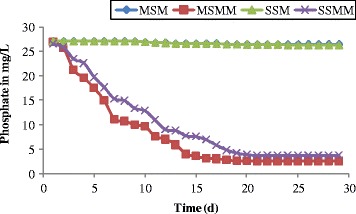
**Changes in phosphate as function of time.**

The changes in nitrate concentration as a function of time are shown in Figure 7. From the Figure 7, the nitrate concentration in the effluent leachate of the blank columns was observed at 3.3 mg/L and it was stable during the 30 days of experimental operation. The reduction of 1.1 mg/L was measured in the microbial column MSMM, due to continuous supply taken by the microbes. The stratified sand microbial column reduces the concentration to 2.1 mg/L at the end day of the experiment due to less growth of microbes. Similar results were reported by Yuliani *et al.* [43]. Likewise, the reduction of phosphates was observed more as 88% in the column MSMM (Figure 8) when compared to the column SSMM. There was a poor reduction in the blank columns from the influent leachate concentration.

**Figure 9:**
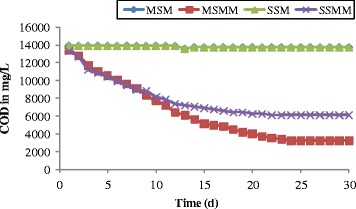
**Changes in COD as function of time.**

The substrate concentration profile was estimated by measuring soluble COD concentrations in each column. Figure 9 shows the COD concentration profile for each column as a function of time. The initial COD concentration of 14000 mg/L was supplied during the experimental operation. In the blank columns (MSM, SSM), there was very little changes in the effluent leachate concentration with a 2% reduction [64,65,76]. In the case of microbial inoculated columns (MSMM, SSMM), the soluble COD (SCOD) reduction in the first 12 days was found to be rapid due to the growth of biofilm accumulation and its continuous intake of synthetic leachate.

Gradual reduction in SCOD was observed for the day 13 to 22 with less nutrient consumption by the microbes. From the day, 23 to 30 the SCOD concentration turns to the steady state with stable consumption by the microbes. After 30 days of the experiment, the reduction in SCOD concentration in MSMM and SSMM was observed 76.19% and 54.21% respectively. The faster consumption of COD as time elapsed implies that microbial growth was continuous throughout the column depth as nutrient and electron acceptors were continuously provided [72,37,77]. When compared to MSMM column, SSMM column achieved a less reduction in the SCOD concentration in the effluent leachate. This may be due to less microbial growth in the coarse sand particles (0.6 mm) of the stratified column.

The reaction rate constant was derived from the pseudo-first-order kinetics plot and is reported in Table 4. From Table 4, the column MSMM shows the highest reaction rate of 0.062 days^−1^ when compared to column SSMM (k = 0.041 days^−1^). This may be due the presence of more microbial attachment in the sand particles.

**Table 4:** **Values of pseudo-first-order constant for different column conditions**

**S. No.**	**Column**	**Reaction rate constant k**_**1**_ **(day** ^**−1**^ **)**	**R** ^**2**^
1.	MSM	0.001	0.140
2.	SSM	0.001	0.140
3.	MSMM	0.062	0.993
4.	SSMM	0.041	0.835

### Biofilm observation by SEM

**Figure 10:**
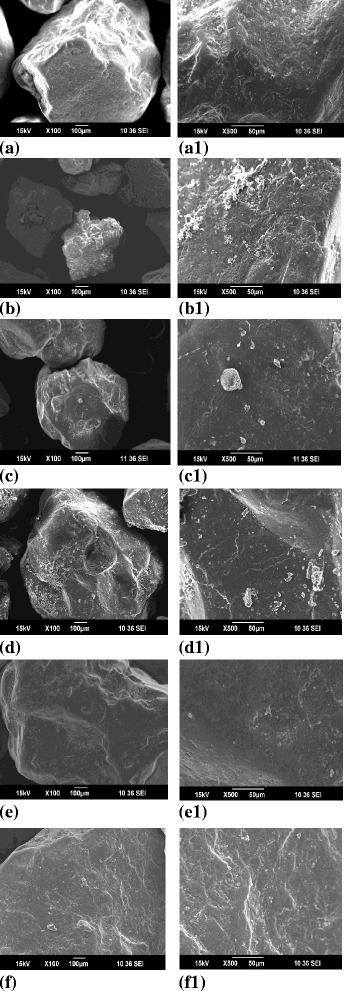
**SEM images of the sand grain surface sampled after 45 days of operation (100×: a - f; 500×: a1 – f1) a, a1.** Mixed sand media without microbes; **b**, **b1**. Mixed sand media with microbes; **c**, **c1**. Stratified fine media without microbes; **d**, **d1**. Stratified fine media with microbes; **e**, **e1**. Stratified coarse media without microbes; **f**, **f1**. Stratified coarse media with microbes.

To understand the clogging phenomenon of biofilm, SEM images were observed for sand samples after the experimental operation. The scanning electron micrographs are presented in Figure 10. Figure 10(a-f) shows a 100 times enlarged SEM image of the sand surface samples to visualize the clogging attachment of an individual grain. Figure 10(a1-f1) shows a 500 times enlarged SEM image of sand surface samples to view the biofilm layer form. From the SEM images, the difference between the sand samples of blank columns and microbial inoculated columns can be seen clearly.

The observations from mixed sand media column were made as follows: Figure 10(a, a1) shows an image of the sand surface scan without clogging. The surface of the sand is smooth. Figure 10(b, b1) shows a picture of the surface scan of the sand after 45 days of clogging, which clearly shows the intensive growth of microbial films [34]. The inoculated bacterium forms several layers of mesh forms of biofilm between sand particles as well as on the sand surface. The formation mesh layers effectively clog the sand pore and result in hydraulic conductivity reduction [49].

The fine sand media were taken from the top of the stratified columns shows that, there was a slight formation of biofilm on the sand surface (Figure 10(c, c1)). This may be due to the less hydraulic conductivity of the fine sand particles used which allows the growth of microbes on the sand surface. Figure 10(d, d1) shows the, clear thick microbial layer on the surface of the sand grain.

The observations of coarse sand samples collected from the stratified column were discussed as follows: From the SEM images it can be seen that there was only a little difference between the blank columns and microbial inoculated columns. Figure 10(e, e1) shows a clear soft surface layer and it was found no microbial growth in the coarse sand media. A very thin layer of biofilm formation visualized on the coarse grain surface of the microbial column (Figure 10(f, f1)). The comparison of fine and coarse sand media concluded that, there was an appreciable formation of biofilm layer occurred only on the fine sand surface.

## Conclusion

In this study, the concept of a biofilm accumulation in a sand column for controlling the leachate migration in the subsurface environment is reported. Synthetic leachate was supplied continuously for the microbial growth to measure the reduction in hydraulic conductivity and organic contaminants of the effluent leachate. Of the four different types of column experiment the following conclusions were made: With scanning electron microscope observation of biofilm, it can be observed that the sand column inoculated with bacteria formed several layers of the biofilm on the sand particles, which resulted in a hydraulic conductivity reduction. There was no appreciable change in the hydraulic conductivity and physico-chemical parameters analyzed from the effluent of blank columns (MSM and SSM). The initial hydraulic conductivity of the column was 2.03 × 10^−2^ cm/s. After 45 days of operation, 98.8% of hydraulic conductivity reduction was observed in the MSMM column with 2.31 × 10^−4^ cm/s. The stratified microbial column (SSMM) reduces the hydraulic conductivity to 1.23 × 10^−3^ cm/s with 93.94%. Hence, the mixed microbial sand column achieves the very good reduction in hydraulic conductivity.

The physico-chemical parameters were also implying the good results in the mixed microbial sand column among all four columns. A proper biological activity was observed from the turbidity and ORP results of MSMM column. Additionally, the reduction in nitrates and phosphates were also observed due to uniformity in the growth of microbes and continuous intake by microbes. In the case of the stratified microbial column, less reduction was taken place in the effluent leachate due to the less microbial growth formation in the coarse sand layer. There is a chance of a detachment of microbes in the coarse layer due to the more space between the sand grain particles.

The organic reduction of influent was estimated by SCOD concentration of each column. The column MSMM shows good reduction at 76.19%, whereas in the column SSMM, 54.21% reduction was achieved. This may be due to the continuous intake of organic substrate by the microorganisms. The pseudo-first-order constant value clearly indicates that the degradation of organic contaminants is due to heavy biofilm accumulation inside the sand column. From the overall results, the biobarrier formed by mixed sand media amended with microbes poses a suitable remedial measure for the reduction of hydraulic conductivity and organic contaminants in the leachate.
